# Comparison of Cecal Microbiota and Performance Indices Between Lean-Type and Fatty-Type Pekin Ducks

**DOI:** 10.3389/fmicb.2022.820569

**Published:** 2022-03-08

**Authors:** Tingshuo Yang, Yong Jiang, Jing Tang, Guobin Chang, Wenming Zhao, Shuisheng Hou, Guohong Chen

**Affiliations:** ^1^College of Animal Science and Technology, Yangzhou University, Yangzhou, China; ^2^Key Laboratory for Animal Genetics & Molecular Breeding of Jiangsu Province, Yangzhou, China; ^3^State Key Laboratory of Animal Nutrition, Institute of Animal Science, Chinese Academy of Agricultural Sciences, Beijing, China

**Keywords:** growth performance, slaughter percentage, plasma biochemical index, cecal microbiota, lean-type, fatty-type, Pekin duck, fat deposition

## Abstract

Fatty-type (FT) Pekin ducks exhibit higher lipid deposition than lean-type (LT) ducks. The gut microbiota plays an important role in modulating fat metabolism. We compared the growth performance, slaughter performance, and cecal microbiota of FT and LT Pekin ducks and analyzed the role of cecal microbiota in lipid deposition in Pekin ducks. A total of 140 1-day-old FT and LT Pekin ducks with similar body weights were randomly assigned to 10 cages, with 14 ducks in each replicate. All ducks were fed commercial diets from 28 to 42 days of age. Results showed that the average body weight and feed intake of FT ducks were higher than those of LT ducks. The breast muscle and eviscerated percentages of LT ducks were higher than those of FT ducks; the abdominal fat and sebum percentages of LT ducks were lower than those of FT ducks at 6 weeks of age (*P* < 0.01). 16S DNA sequencing of the cecal microbiota revealed that the bacterial abundance differed between FT and LT ducks at 4 and 6 weeks of age. The abundance of Firmicutes was higher, while that of Fusobacteria and *Fusobacterium* was lower in LT ducks than in FT ducks at 4 weeks of age. The abundance of Spirochaetes was higher, while that of Firmicutes and Bacteroides was lower in LT ducks than in FT ducks at 6 weeks of age. The abundance of Spirochaetes and Brachyspira in LT ducks was higher at 6 weeks than at 4 weeks of age. Interestingly, the abundance of Firmicutes and Bacteroides in FT ducks was higher at 6 weeks of age than at 4 weeks of age, while that of Fusobacteria and *Fusobacterium* was lower at 6 weeks than at 4 weeks of age. Linear discriminant analysis effect size analysis showed that *Spirochaetes*, *Brachyspira*, *Alistipes*, *Campylobacter*, *Megamonas*, *Butyricicoccus*, and Fusobacteria may be involved in the fat metabolism pathway as specific markers. We reveal the differences in microbial abundance in the cecal microbiota between FT and LT Pekin ducks and provide an insight into the role of cecal microbiota in lipid deposition in Pekin ducks.

## Introduction

Pekin duck is a popular indigenous Chinese duck species characterized by rapid growth, excellent fattening, and a high reproductive rate. Additionally, roasted Pekin duck is a globally recognized dish that requires Pekin ducks with increased fat deposition, referred to as fatty-type (FT) Pekin ducks. With an increasing demand for meat production, a new line of Pekin ducks with a low subcutaneous fat content and high muscle percentage (lean type, LT) is required for consumption. Therefore, in recent decades, continuous genetic selection has been used to breed LT Pekin ducks in China (Hou and Hu, 2005). FT and LT Pekin ducks exhibit significant differences in meat production, sebum, and abdominal fat contents (Zheng et al., 2014). A previous study showed that under conditions of Thr deficiency, both FT and LT ducks had similar body weights at 35 days of age; however, FT ducks had higher abdominal fat and sebum percentages and a lower hepatic lipid content (Jiang et al., 2020). To explore the underlying mechanism of the different lipid profiles between FT and LT Pekin ducks, we performed transcriptome analysis to compare differentially expressed genes in the liver, sebum, and abdominal fat. We previously found that, compared to LT ducks, FT ducks exhibit a high expression of genes involved in lipid synthesis in the liver and sebum and low expression of genes involved in lipid degradation in abdominal fat (unpublished data).

The gut microbiota is a key factor in lipid deposition. A healthy diet influences the degradation potential of intestinal microbial plant polysaccharides, lipid metabolism into short-chain fatty acids and pectin metabolism, and is positively correlated with producers of dietary fiber metabolites and short-chain fatty acids (Wang D.D. et al., 2021). The combined analysis of the oral and gut microbiome and lipid metabolome of COVID-19 patients and recovered patients showed that lipid molecules were closely associated with changes in the oral and gut microbial communities (Ren et al., 2021). The mouse gut microbiome exhibits specific changes during the progression of perimenopausal atherosclerosis and is significantly associated with lipid metabolites, and estrogen supplementation may have beneficial effects on gut bacteria and lipid metabolism (Meng et al., 2021). In terms of the proportion of intestinal microbiota, obese humans show a low abundance of Bacteroidetes and a high abundance of Firmicutes compared to lean humans, with the relative proportion of Bacteroidetes increasing with weight loss (Ley et al., 2007). Gut microbial shifts were observed across pre-pregnancy, gestation, and lactation in lean and high-fat/sucrose diet-induced obese rats. *Lactobacillus* and *Bifidobacterium* spp. in the gut microbiota of lean rats were present in higher levels than in obese rats. *Clostridium* cluster XI and *Clostridium* cluster I abundance was higher in obese rats than in lean rats (Paul et al., 2018). Additionally, obese horses have more diverse gut microbiome communities, with a higher relative abundance of Firmicutes and lower numbers of Bacteroidetes and Actinobacteria than those in lean horses; *Butyrivibrio* spp., Prevotellaceae, and *Blautia* spp. are positively associated with obesity in the host (Biddle et al., 2018). Furthermore, the gut microbiota and its metabolic products act as a bridge between the diet and host and maintain body health (Nicholson et al., 2012). Their main functions include mediating physiological metabolic activities and the consumption, storage, and redistribution of energy. Transferring fecal microbiota from adult chickens with high body weight into one-day-old chickens improved growth performance and fat metabolism in liver by remodeling the gut microbiota (Zhang et al., 2021). Gut microbes can degrade polysaccharides and oligosaccharides into simple metabolites, such as short-chain fatty acids, which function as energy sources for tissues and cells, can enhance intestinal barrier function, and promote glucose and lipid biosynthesis and metabolism in the liver (Kelly et al., 2015). In addition, intestinal microorganisms can regulate lipid biosynthesis by affecting fatty acid metabolism (Bjursell et al., 2011). Lactic acid, acetic acid, and butyric acid, produced by *Lactobacillus* and *Bifidobacterium* as the main metabolites (Picard et al., 2005), are important energy sources for intestinal epithelial cells and regulate the growth and differentiation of intestinal epithelial cells (Boffa et al., 1992; Bugaut and Bentéjac, 1993). Therefore, changes in the gut microbial ecology can lead to significant changes in body weight and the energy balance (Flegal and Troiano, 2000). The difference of microbiota improved the growth performance of broilers, which may be due to the difference in enteric microbiota between cage and floor rearing systems (Wang L.D. et al., 2021). The caeca microbial communities of broiler chicken were more diverse in comparison to ilea. Distinction in functions and roles of gut microbiota such as gene pathways related to nutrient absorption (e.g., sugar and amino acid metabolism), and bacterial proliferation and colonization (e.g., bacterial motility proteins, two-component system, and bacterial secretion system) were observed in caeca, respectively (Barnes et al., 1972; Such et al., 2021; Xiao et al., 2021).

In addition, the degradation products of dietary nutrients, such as monosaccharides and short-chain fatty acids (Cho et al., 2012; Zhao et al., 2018), stimulate triglyceride synthesis in the liver and promote lipid deposition in the adipose tissue (Bäckhed et al., 2004). Intestinal microorganisms can also promote fat deposition by influencing the expression of genes related to lipid metabolism in the host (Tilg and Moschen, 2014). Bacterial strains are a major internal factor promoting the development of intestinal microbiota in pigs and mice (Ley et al., 2005; Bian et al., 2016). In one study, the relative abundance of Bacteroides in fatty mice was lower than that in lean mice, whereas the proportion of Firmicutes was higher. Differences in lipid deposition between fat and lean mice were not attributed to different feed consumption, but rather in part to differences in gut microbes (Ley et al., 2005). Therefore, the differences in lipid deposition between FT and LT ducks may be related to the cecal microbiome. This study was conducted to compare the predominant microbial community between FT and LT Pekin ducks *via* random shotgun sequencing of their cecal microbial DNA in the V3 and V4 domains and investigate the role of cecal microbiomes in lipid deposition in Pekin ducks.

## Materials and Methods

### Experimental Design and Diets

A completely randomized design involving two duck strains (FT Pekin ducks and LT Pekin ducks) was used. At 1 day of age, 280 Pekin ducks [140 FT ducks (high-sebum percentage) and 140 LT ducks (low-sebum percentage)] were assigned to 20 cages with 14 ducks per cage. All birds were housed in raised wire floor pens (200 × 100 × 40 cm) from birth to 6 weeks of age, and their feed intake was recorded from 28 to 42 days of age. The cages were equipped with nipple drinkers and tubular feeders, and the house was maintained under constant light. The room temperature was maintained at 30°C from 1 to 3 days of age; it was then gradually reduced to 20°C until 21 days of age and maintained until the end of 6 weeks. Feed pellets and water were provided *ad libitum*. All experimental ducks were fed a commercial diet.

Commercial duckling compound feed at 1 to 21 days of age was purchased from Hope Feed Co., Ltd., (Fangshan District, Beijing, China), and the main raw materials were corn, soybean meal, corn gluten meal, stone powder, calcium bicarbonate, manganese sulfate, vitamin A, and vitamin B2. Product guarantee value composition analysis showed the following values: crude protein, ≥19.0%; crude ash, ≤8.0%; crude fiber, ≤6.0%; calcium, 0.5–1.5%; phosphorus, ≥0.3%; sodium chloride, 0.2–0.8%; methionine, ≥0.3%; and moisture, ≤14.0%. Commercial duck compound feed at 22 to 42 days of age was purchased from Hope Feed Co., Ltd., (Fangshan District, Beijing, China), and the main raw materials were: corn, corn gluten powder, stone powder, calcium bicarbonate, manganese sulfate, vitamin A and vitamin B2. Product guarantee value composition analysis showed the following values: crude protein, ≥16.0%, ash, ≤9.0%, crude fiber, ≤6.0%, calcium, 0.9–1.5%, total phosphorus, ≥0.3%, sodium chloride, 0.2–0.8%, methionine, ≥0.2%, moisture, ≤14.0%.

### Performance Parameters, Sampling, and Data Processing

At 4 and 6 weeks of age, the body weight (BW) and feed intake of ducks in each cage were recorded after a 12 h fast. At 6 weeks of age, two ducks were selected according to the average body weight (ABW) of the cage and then weighed. At the initial age of the 14 one-day-old ducks per cage, two ducks per cage were slaughtered at 4 weeks of age and two ducks per cage at 6 weeks of age. Blood samples were collected from each bird, *via* wing vein puncture, in 5 mL heparinized vacuum-tube syringes with stainless-steel needles. The blood samples were centrifuged at 1000 × *g* for 15 min at 4°C to separate the plasma. The plasma samples were stored at −20°C until analysis of biochemical parameters. The ducks were immediately sacrificed *via* cervical dislocation after carbon dioxide anesthesia. The extracted breast muscles, thigh muscle, abdominal fat, and sebum were used to calculate slaughter performance. Cecum contents were collected into 1.5 mL tubes, quickly frozen in liquid nitrogen, and then stored at −80°C.

### Plasma Parameters

Ten cages were used for each of the two Peking duck strains. During weeks 4–6, blood samples were collected from three ducks in each cage according to the ABW of the remaining ducks. After centrifuging the blood samples, the upper transparent, faint yellow plasma was collected. The plasma was divided into 1.5 mL centrifuge tubes and stored at −20°C; the samples were thawed at room temperature before analysis. The plasma concentrations of cholesterol (CHOL), triglyceride (TG), low-density lipoprotein cholesterol (LDL-C), and high-density lipoprotein cholesterol (HDL-C) were measured using an automatic analyzer (7080, Hitachi, Tokyo, Japan) and commercial kits (Maccura Biotechnology Co., Ltd., Chengdu, Sichuan Province, China).

### Preprocessing of Sequencing Data

Genomic DNA of the samples was extracted using the cetyltrimethyl ammonium bromide or sodium dodecyl sulfate methods, and the purity and concentration of DNA were determined using agarose gel electrophoresis. An appropriate amount of sample DNA was diluted to 1 ng/L with sterile water and used as a template. Based on selection of the sequencing region, a specific primer with a barcode, Phusion^®^ high-fidelity PCR Master Mix with GC Buffer (New England Biolabs, Ipswich, MA, United States), and high-fidelity Taq polymerase were used for polymerase chain reaction (PCR) to ensure amplification efficiency and accuracy. The V3-V4 primer pair of 16S is 341F-806R (CCTAYGGGRBGCASCAG-GGACTFACNNGGGTATCTAAT).

Polymerase chain reaction products were detected using 2% agarose gel electrophoresis. Equal amounts of samples were mixed according to the concentrations of PCR products; after full blending, 2% agarose gel electrophoresis was performed to detect the PCR products. The target bands were recovered using a gel recovery kit (Qiagen, Hilden, Germany). The TruSeq^®^ DNA PCR-Free Sample Preparation Kit was used to construct the library, which was quantified using a Qubit and quantitative PCR. After qualified control of the library, a NovaSeq6000 platform (Illumina, San Diego, CA, United States) was used for on-board sequencing. Ten samples were used from the LT and FT groups, except for the FT group at 4 weeks of age, as one sample was contaminated.

### Statistical Analysis of the Sequencing Data

Based on barcoding and PCR amplification of the sequences from all of the sample data, the sequences were truncated using FLASH (Magoč and Salzberg, 2011). The raw reads were spliced using QIIME (Caporaso et al., 2011), and quality control (Rognes et al., 2016) was performed to obtain the final valid data. The following operations were used: (A) tag interception: truncate raw tags from the first low-quality base site with a continuous low-quality value (default quality threshold is ≤19) number of bases reaching the set length (default length was 3); (B) tag length filtering: the tag data set obtained after the tags were intercepted, further filtering out tags with a continuous high-quality base length of less than 75% of the tag length. The tags obtained after these steps were processed to remove chimeric sequences. Tag sequence was compared with sequences in the species annotation database to detect the chimeric sequence, and the chimeric sequence was removed to obtain the final effective tags.

The UPARSE software (Haas et al., 2011) was used to cluster all effective tags in all samples, and by default, sequences were clustered into operational taxonomic units (OTUs) with 97% consistency (identity) (Wang et al., 2007). To annotate the OTUs, Mothur and the SILVA 132 database (Edgar, 2013), along with SSUrRNA database species annotation analysis (setting threshold of 0.8–1), were used to acquire taxonomic information at each classification level: kingdom (community), phylum (door), class (class), order (mesh), family (family), genus (genus), and species (kind of) statistics community composition of each sample (Quast et al., 2013). MUSCLE software was used for fast multi-sequence alignment to homogenize the data from each sample, after which diversity analyses were performed based on sequence alignments. OTU-level alpha diversity indices, such as the Chao richness estimator, ACE metric (abundance-based coverage estimator), Shannon diversity index, and Simpson index, were used to investigate the richness and evenness of microbial communities among the four groups and were calculated using the OTU table in QIIME. Linear discriminant analysis (LDA) effect size (LEfSe) analysis was performed to reveal the significant ranking of abundant modules in the experimental group. A size-effect threshold of 4.0, based on the logarithmic LDA score, was used for discriminative functional markers. Tax4Fun function prediction maps were used to annotate prokaryotic genome-wide function information to the SILVA database for functional annotation.

### Data Analysis

Data were analyzed using the SAS software (version 9.4, SAS Institute, Cary, NC, United States) for *t*-tests, and the results were expressed as mean ± standard deviation. The level of statistical significance was set at *P* < 0.05. Multiple comparative analyses were performed using the SPSS 22.0 software (SPSS, Inc., Chicago, IL, United States).

The control group was labeled as L.WK4, which comprised 4-week-old LT Peking ducks; F.WK4, which comprised 4-week-old FT Peking ducks; L.WK6, which comprised 6-week-old LT Peking ducks; and F.WK6, which comprised 6-week-old FT Peking ducks.

The alpha diversity index was described as follows. The Chao and ACE indices were used to calculate the abundance of bacteria. The Shannon and Simpson indices were used to calculate the diversity of bacterial communities. The R software (The R Group for Statistical Computing, Vienna, Austria) was used to analyze differences in the alpha diversity index between groups. Difference analysis between groups based on the alpha diversity index was conducted using parametric and non-parametric tests. If there were only two groups, *t*-test and Wilcoxon test were used; for more than two groups, Tukey test and Agricolae package Wilcox test were used.

Beta variation calculation methods included the unweighted pair group method with arithmetic mean (UPGMA), principal component analysis (PCA), and partial least squares discriminant analysis (PLS-DA). The Qiime software (version 1.9.1) was used to calculate the UniFrac distance and build the UPGMA sample cluster tree. PCA and PLS-DA diagrams were drawn using the R software (version 2.15.3). The Ade4 and GGploT2 packages in R were used for PCA. The Mixomics package of R was used for PLS-DA. Furthermore, the R software was used to analyze differences in the beta diversity index between groups. Parametric and non-parametric tests were also conducted. If there were only two groups, *t*-test and Wilcoxon test were used; for more than two groups, Tukey test and Agricolae package Wilcox test were used.

Linear discriminant analysis effect size analysis was performed using the LEfSe software, and the default value of the LDA score was 4. First, the non-parametric Kruskal-Wallis rank sum was used to detect species with significantly different abundance between different groups, and the Wilcoxon rank sum was used to determine the consistency of differences in the different subgroups of different species from the previous step; finally, LDA was used to estimate the magnitude of the effect of the abundance of each component (species) on the differential effect.

ANOSIM is a non-parametric test used to evaluate whether the difference between groups is significantly greater than the difference within the group to determine whether the grouping is meaningful (Chapman and Underwood, 1999). ANOSIM was conducted using the R vegan package ANOSIM function, and a significance test of the difference between groups was performed based on the rank of the Bray-Curtis distance value.

Tax4Fun functional prediction was performed using the nearest neighbor method based on the minimum 16S rRNA sequence similarity. Specifically, we extracted the 16S rRNA gene sequence of the whole genome of prokaryotes from the Kyoto Encyclopedia of Genes and Genomes (KEGG) database and compared the results with the SILVA SSU Ref NR database (BLAST bit score >1500), using the BLASTN algorithm, to establish a correlation matrix. The whole-genome functional information of prokaryotes annotated using UProC and PAUDA in the KEGG database corresponded to that in the SILVA database to realize functional annotation of the SILVA database. SILVA database sequence was used as a reference to cluster OTUs and obtain functional annotation information.

Regularized canonical correlation analysis is used to highlight correlations between datasets. MixOmics is an R package used for statistical analyses of large biological datasets. MixOmics focuses on data visualization to better interpret the results. Correlation circle plots and clustered image maps were used as input by-products of the integrative approaches implemented in the MixOmics package (González et al., 2012).

## Results

### Growth Performance

Fatty-type ducks had higher (*P* < 0.01) initial BW at 4 weeks of age, ABW at 6 weeks of age, and average daily feed intake from 4 to 6 weeks than LT ducks (Table 1). However, the feed/gain and average daily gain between FT and LT ducks were not significantly different (*P* > 0.05).

**TABLE 1:** Growth performance of lean-type and fatty-type Pekin ducks from 4 to 6 week of age.

Strains	ABW at 4 week, g	ABW at 6 week, g	ADFI, g	ADG, g	F/G, (g/g)
Lean^1^	2487.3 ± 108.5^b^	3131.2 ± 96.48^b^	271.4 ± 25.27^b^	55.65 ± 15.19	5.27 ± 1.75
Fatty^1^	2712.2 ± 59.5^a^	3278.6 ± 61.07^a^	322.1 ± 10.8^a^	54.79 ± 8.75	6.20 ± 1.01
*P*-value	<0.0001	0.0017	< 0.0001	0.8891	0.2993

*Different shoulder marks of lowercase letters mean significant difference (P < 0.05). ABW, average body weight; ADFI, average daily feed intake; ADG, the average daily gain; F/G, Feed/Gain; g, Gram.*

*^1^Data represent the means of 8 replicate cages (n = 8).*

### Carcass Traits

The results of slaughter performance of LT and FT Pekin ducks at 6 weeks of age are listed in Table 2. The percentages of breast muscle and evisceration in LT Pekin ducks were significantly higher than those in FT ducks; however, the percentages of abdominal fat and sebum in LT ducks were lower than those in FT ducks (*P* < 0.01). There were no significant differences in the percentage of leg muscle and eviscerated yield (*P* > 0.05).

**TABLE 2:** Slaughter performance of lean-type and fatty-type Pekin ducks at 6 weeks of age (%).

Strains	Abdominal fat	Sebum	Breast muscle	The leg muscle	Yield of carcass	Eviscerated yield
Lean^1^	6.37 ± 0.96^b^	25.22 ± 2.04^b^	14.95 ± 1.33^a^	11.29 ± 1.12	86.41 ± 0.78	74.09 ± 1.20^a^
Fatty^1^	7.75 ± 1.05^a^	32.48 ± 3.85^a^	11.41 ± 1.72^b^	10.89 ± 1.13	86.54 ± 0.83	72.92 ± 1.48^b^
*P*-value	<0.0001	<0.0001	<0.0001	0.2697	0.6034	0.0091

*Different shoulder marks of lowercase letters mean significant difference (P < 0.05), different capital letters mean extremely significant difference (P < 0.01), and the same or no letters mean insignificant difference (P > 0.05).*

*^1^Data represent the means of 8 replicate cages (n = 8).*

### Blood Biochemical Indices

The plasma CHOL, HDL-C, and LDL-C contents of FT Pekin ducks at 4 weeks of age were significantly higher than those of LT Pekin ducks (*P* < 0.01), but there was no significant difference in the plasma TG content (*P* > 0.05). In contrast, the plasma CHOL, TG, HDL-C, and LDL-C contents of FT Pekin ducks at 6 weeks of age were significantly higher than those of LT Pekin ducks (Table 3).

**TABLE 3:** Comparison of plasma biochemical indexes between fatty-type and lean-type Pekin duck at 4 and 6 weeks of age.

Weeks of age	Items	Lean-type	Fatty-type	*P*-value
4	CHOL (μmol/L)	5.17 ± 1.45^b^	6.80 ± 1.20^a^	<0.0001
	TG (mmol/L)	0.30 ± 0.10	0.34 ± 0.12	0.2177
	HDL-C (μmol/L)	2.94 ± 0.73^b^	3.57 ± 0.65^a^	0.0008
	LDL-C (μmol/L)	1.29 ± 0.47^b^	2.15 ± 0.64^a^	<0.0001
6	CHOL (μmol/L)	4.69 ± 0.64^b^	5.96 ± 0.57^a^	<0.0001
	TG (mmol/L)	0.39 ± 0.08^b^	0.54 ± 0.11^a^	<0.0001
	HDL-C (μmol/L)	2.76 ± 0.36^b^	3.31 ± 0.33^a^	<0.0001
	LDL-C (μmol/L)	1.12 ± 0.21^b^	1.59 ± 0.29^a^	<00001

*Different shoulder marks of lowercase letters mean significant difference (P < 0.05), different capital letters mean extremely significant difference (P < 0.01), and the same or no letters mean insignificant difference (P > 0.05).*

*^1^Data represent the means of 8 replicate cages (n = 8). CHOL, cholesterol; TG, triglyceride; HDL-C, high density lipoprotein cholesterol; LDL-C, low density lipoprotein cholesterol.*

### 16S rRNA Sequences of Lean-Type and Fatty-Type Pekin Ducks

**FIGURE 1:**
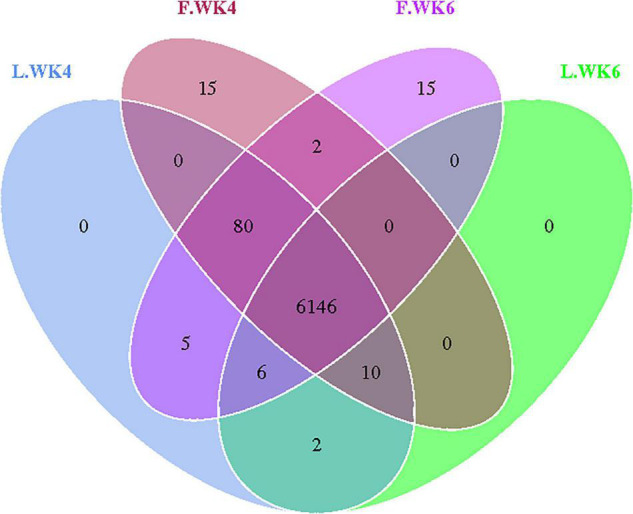
Effects of different age and strain on cecal microbial OUT. Venn diagrams for microbial OTU compositions. Control group: L.WK4, Four-week-old lean-type Pekin duck; F.WK4, Four-week-old fatty-type Pekin duck; L.WK6, Six-week-old lean-type Pekin duck; F.WK6, Six-week-old fatty-type Pekin duck.

We amplified the DNA from the cecal microbiota of ducks at 4 and 6 weeks of age (*n* = 10). Paired-end sequencing was performed using the Illumina Nova sequencing platform to construct a PCR-free library. Through read splicing, an average of 94,127 tags was measured per sample. After quality control, 89,491 effective data points were obtained on an average. A 97% consistency (identity) was used to cluster the sequences into OTUs, generating 1,108 OTUs based on 97% species similarity, after which species annotation was performed using OTU sequences from the SILVA 132 database. The 1,108 OTUs were classified into 20 phyla, 29 classes, 51 orders, 90 families, and 178 genera (Table 4). A Venn diagram was used to show the common and unique OTUs among the four groups (Figure 1). We identified 6,249 and 6,253 OTUs in the LT and FT ducks at 4 weeks of age, respectively, and 6,254 and 6,163 OTUs in the LT and FT ducks at 6 weeks of age, respectively.

**TABLE 4:** The quantity of taxons of cecal microbiota of lean-type and fatty-type Pekin ducks at 6 weeks of age.

Taxon	Quantity
Kingdom	2
Phylum	20
Class	29
Order	51
Family	90
Genus	178
Species	140

### Cecal Microbiota Diversities in Lean-Type and Fatty-Type Pekin Ducks

**FIGURE 2:**
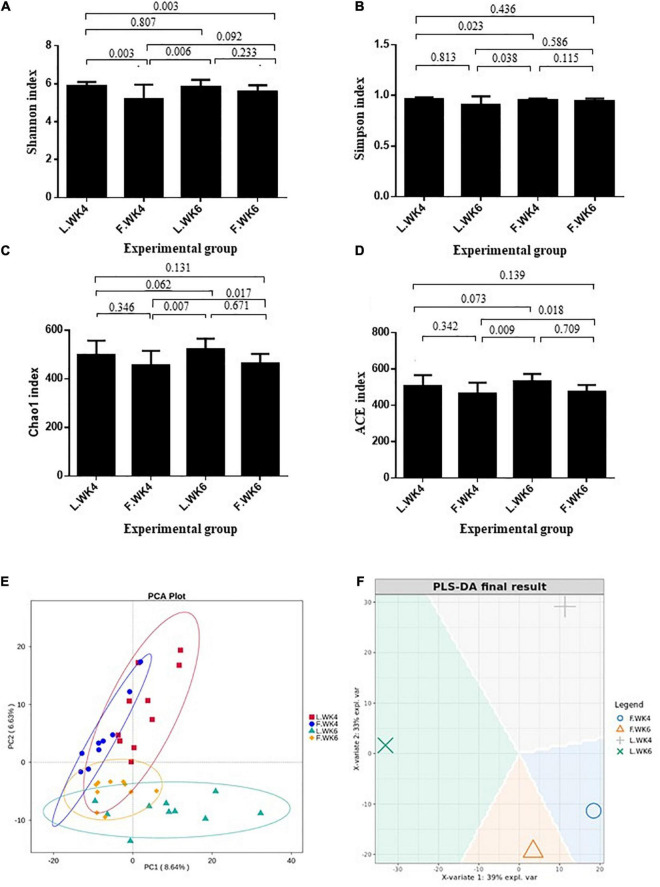
Effects of different age and strain on the diversities in the cecal microbial. **(A)** Shannon index. **(B)** Simpson index. **(C)** Chao1 index. **(D)** ACE index. **(E)** The principal component analysis. PCA plot about the cecal microbiota. **(F)** PLS-DA sample plot with confidence ellipse plots. Control group: L.WK4, Four-week-old lean-type Pekin duck; F.WK4, Four-week-old fatty-type Pekin duck; L.WK6, Six-week-old lean-type Pekin duck; F.WK6, Six-week-old fatty-type Pekin duck (data are mean ± SD).

**FIGURE 3:**
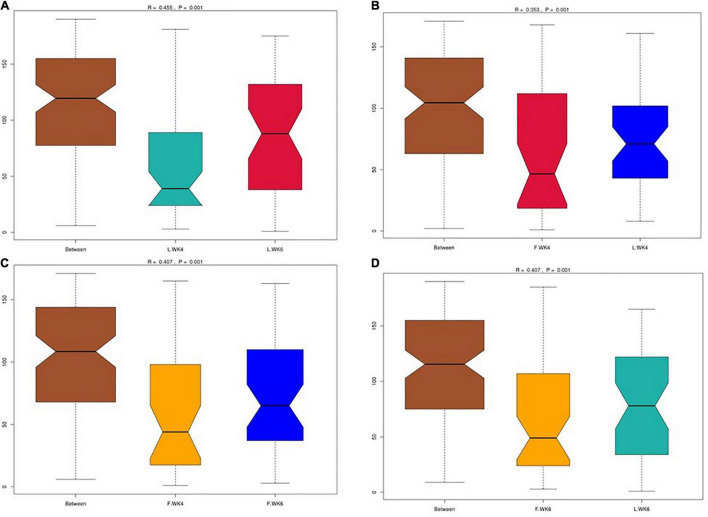
**(A)** Box plot of inter-group and intra-group beta distance (ANOSIM analysis) of L.WK4 and L.WK6. **(B)** Box plot of inter-group and intra-group beta distance (ANOSIM analysis) of F.WK4 and L.WK4. **(C)** Box plot of Inter-group and Intra-group Beta distance (ANOSIM analysis) of F.WK4 and F.WK6. **(D)** Box plot of inter-group and intra-group beta distance (ANOSIM analysis) of F.WK6 and L.WK6. L.WK4, four-week-old lean-type Pekin duck; F.WK4, four-week-old fatty-type Pekin duck; L.WK6, six-week-old lean-type Pekin duck; F.WK6, six-week-old fatty-type Pekin duck.

Alpha diversity was analyzed to determine the dynamics of the cecal microbiota of LT and FT ducks at 4 and 6 weeks of age. The Shannon and Simpson indices of LT ducks were higher than those of FT ducks at 4 weeks of age (Figures 2A,B). The Chao1 index of LT ducks was higher than that of FT ducks at 6 weeks of age (Figure 2C). At 6 weeks of age, the ACE index of LT ducks was higher than that of LT ducks at 4 weeks of age and FT ducks at 6 weeks of age (Figure 2D). Furthermore, beta variation was calculated to evaluate the variations in cecal microbiota community. PCA revealed significant differences in LT ducks at 4 and 6 weeks of age and in FT ducks at 4 and 6 weeks of age (Figure 2E). Supervised analysis with PLS-DA focused on discrimination of the four groups (Figure 2F). The intergroup and intragroup β distances are shown in a box plot in Figure 3. The results revealed extremely significant differences in bacterial communities (*P* < 0.01) between the L.WK4 and L.WK6 groups (Figure 3A), between the F.WK4 and L.WK4 groups (Figure 3B), between the F.WK4 and F.WK6 groups (Figure 3C), and between the F.WK6 and L.WK6 groups (Figure 3D). The difference between groups was significantly greater than that within the group. Thus, this grouping was meaningful.

### Differences in Community Diversity Between Lean-Type and Fatty-Type Pekin Ducks

**FIGURE 4:**
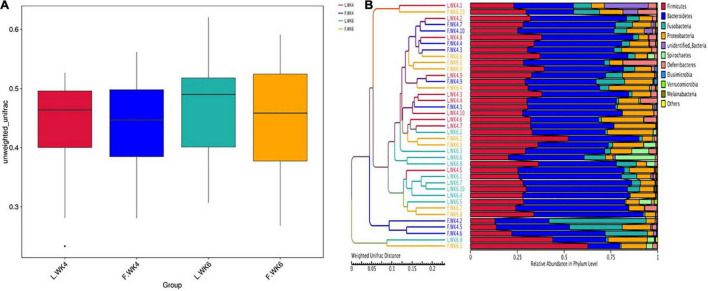
Comparison of cecal microbial community structure between lean-type and fatty-type Pekin duck. **(A)** The beta-diversity index (weighted UniFrac distance) was not significant among the 4 groups (*P* > 0.05). The box shows the quartiles above and below the median, with a dark line at the center of the box denoting the median and black dots outside the box showing the outlier. **(B)** The UPGMA Cluster Tree displaying the relative abundance of predominant bacteria at the phylum level in each group (weighted UniFrac distance). L.WK4, Four-week-old lean-type Pekin duck; F.WK4, Four-week-old fatty-type Pekin duck; L.WK6, Six-week-old lean-type Pekin duck; F.WK6, Six-week-old fatty-type Pekin duck.

The beta-diversity index of the OTU community structure between samples showed a high level of similarity among the four groups (L.WK4, F.WK4, L.WK6, and F.WK6) despite the difference in the genetic backgrounds (Figure 4A). The UPGMA clustering tree based on Weighted Unifrac distance revealed that the dominant bacterial phyla in the four groups were Firmicutes, Bacteroidetes, Fusobacteria, Proteobacteria, Spirochaetes, Deferribacteres, Elusimicrobia, Verrucomicrobia, and Melainabacteria (Figure 4B).

### Differences in Microbial Community Composition Between Lean-Type and Fatty-Type Pekin Ducks

**FIGURE 5:**
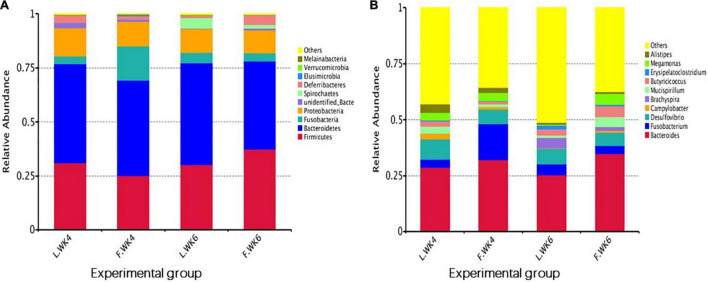
The top 10 relative abundance of microbiota community (level phylum and level genera) indicate by histogram. **(A)** The top 10 phylum with the highest abundance. **(B)** The top 10 genera with the highest abundance. L.WK4, Four-week-old lean-type Pekin duck; F.WK4, Four-week-old fatty-type Pekin duck; L.WK6, Six-week-old lean-type Pekin duck; F.WK6, Six-week-old fatty-type Pekin duck.

The top 10 phyla and top 10 genera in terms of relative abundance of the cecal bacteria in LT and FT Pekin ducks are shown in Figure 5. Firmicutes and Bacteroidetes were the most prevalent phyla in both LT and FT Pekin ducks, followed by Fusobacteria and Proteobacteria. Fusobacteria and Proteobacteria accounted for 75.56 and 18.27% of the total sequence, respectively. Other phyla included Spirochaetes, Deferribacteres, Elusimicrobia, Verrucomicrobia, and Melainabacteria. Only 0.25% of sequences were unclassified at the phylum level. At the genus level, *Bacteroides* was dominant in FT and LT ducks, whereas *Fusobacterium*, *Desulfovibrio*, *Campylobacter*, *Brachyspira*, *Mucispirillum*, *Butyricicoccus*, *Erysipelatoclostridium*, *Megamonas*, and *Alistipes*. These genera accounted for more than 41.97% of the total sequences. These results suggest that the microbial community is influenced by strain and age. The abundance of the top 10 phyla in different groups and the corresponding *P*-values are listed in Supplementary Table 1. The abundance of the top 10 genera in different groups and the *P*-values are listed in Supplementary Table 2.

**FIGURE 6:**
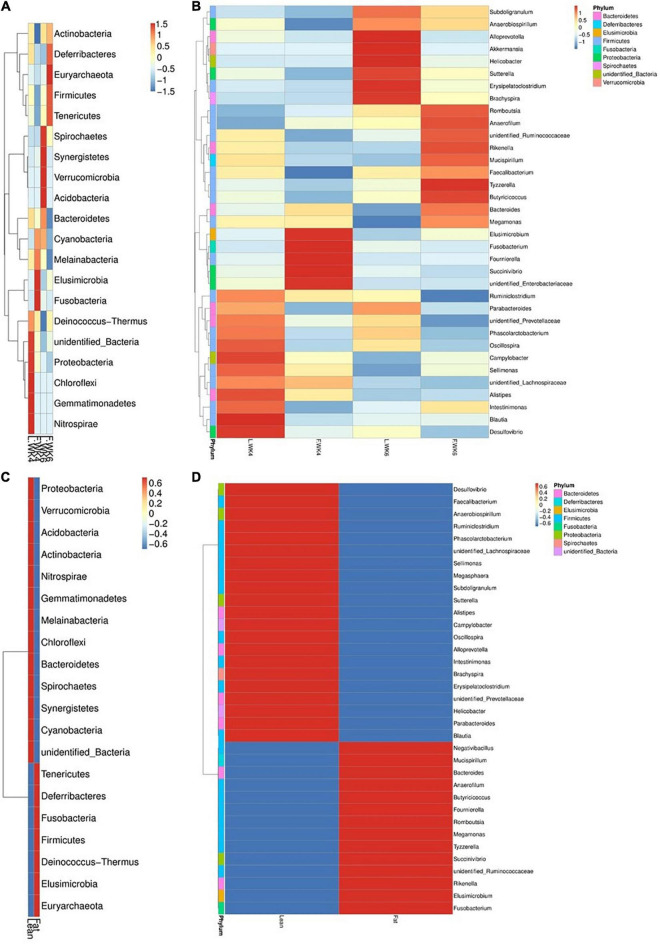
Phylum and genus level OTU analysis taxa heatmap cluster group. **(A)** Heatmap of the top 20 most abundant phyla. **(B)** The top 35 most abundance genera in microbial samples in each group. **(C)** Heatmap of the top 20 most abundant phyla. **(D)** The top 35 most abundance genera in two kinds of strains of microbial samples. The phylum and genera with higher abundance are shown in red, whereas those with lower abundance are shown in blue. L.WK4, Four-week-old lean -type Pekin duck; F.WK4, Four-week-old fatty-type Pekin duck; L.WK6, Six-week-old lean-type Pekin duck; F.WK6, Six-week-old fatty-type Pekin duck.

The top 20 bacterial phyla and top 35 genera in terms of relative abundance differed among the four groups. The abundance of some phyla and genera differed between LT and FT ducks. The most abundant phyla were Nitrospirae, Gemmatimonadetes, Chloroflexi, and Proteobacteria at 4 weeks of age and Spirochaetes, Synergistetes, Verrucomicrobia, and Acidobacteria at 6 weeks of age. Elusimicrobia and Fusobacteria were the most abundant phyla in FT ducks at 4 weeks of age and Euryarchaeota at 6 weeks of age. FT ducks showed a lower abundance of Actinobacteria at 4 weeks of age and Bacteroidetes and Melainabacteria at 6 weeks of age (Figure 6A).

At the genus level, the abundance of *Blautia* and *Desulfovibrio* in LT ducks at 4 weeks of age was higher than that in the other three groups. The abundance of *Elusimicrobium*, *Fusobacterium*, *Fournierella*, and *Succinivibrio* in FT ducks at 4 weeks of age was higher than that in the other three groups. Moreover, the abundance of *Faecalibacterium* in FT ducks at 4 weeks of age was lower than that in the other three groups. The abundance of *Alloprevotella*, *Akkermansia*, *Helicobacter*, *Erysipelatoclostridium*, and *Brachyspira* was higher in LT ducks at 6 weeks of age than in the other three groups, and *Megamonas* abundance in LT ducks at 6 weeks of age was lower than that in the other three groups. The abundance of *Tyzzerella* and *Ruminiclostridium* in FT ducks at 6 weeks of age was higher and lower, respectively, than that in the other three groups (Figure 6B).

At the phylum level, the abundance of Proteobacteria, Verrucomicrobia, Acidobacteria, Actinobacteria, Nitrospirae, Gemmatimonadetes, Melainabacteria, Chloroflexi, Bacteroidetes, Spirochaetes, Synergistetes, and Cyanobacteria in LT ducks was higher than that in FT ducks. The abundance of Tenericutes, Deferribacteres, Fusobacteria, Firmicutes, Deinococcus–Thermus, Elusimicrobia, and Euryarchaeota in FT ducks was higher than that in LT ducks (Figure 6C).

At the genus level, the abundance of *Desulfovibrio*, *Faecalibacterium*, *Anaerobiospirillum*, *Ruminiclostridium*, *Pha scolarctobacterium*, *Sellimonas*, *Megasphaera*, *Subdoligranulum*, *Sutterella*, *Alistipes*, *Campylobacter*, *Oscillospira*, *Alloprevotella*, *Intestinimonas*, *Brachyspira*, *Erysipelatoclostridium*, *Helicobacter*, *Parabacteroides*, and *Blautia* in LT ducks was higher than that in FT ducks. In contrast, the abundance of *Negativibacillus*, *Mucispirillum*, *Bacteroides*, *Anaerofilum*, *Butyricicoccus*, *Four nierella*, *Romboutsia*, *Megamonas*, *Tyzzerella*, *Succinivibrio*, *Rikenella*, *Elusimicrobium*, and *Fusobacterium* in LT ducks was lower than that in FT ducks (Figure 6D).

**FIGURE 7:**
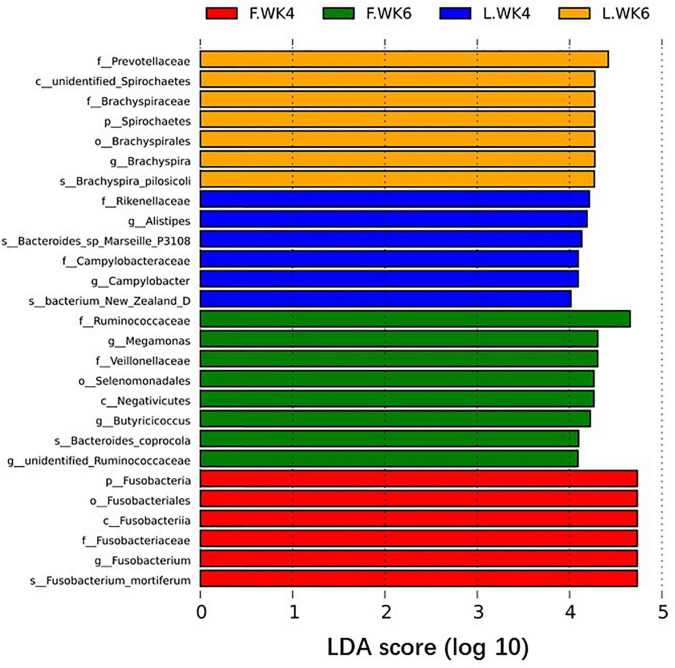
Biomarkers of discriminative bacteria (from phylum to genus) in different temperature groups identified by LEfSe analysis (LDA score ≥ 4). The length of the bar chart represents the influence size of the different species (i.e., LDA score). L.WK4, Four-week-old lean-type Pekin duck; F.WK4, Four-week-old fatty-type Pekin duck; L.WK6, Six-week-old lean-type Pekin duck; F.WK6, Six-week-old fatty-type Pekin duck.

Linear discriminant analysis effect size analysis identified 27 OTUs as biomarkers at a threshold of LDA score >4, with significant differences in the cecal microbiota in LT and FT ducks at 4 and 6 weeks of age (*P* < 0.05). Analysis at the phylum and genus levels revealed two biomarker bacteria (*Spirochaetes* and *Brachyspira*) in LT ducks at 6 weeks of age, two biomarker bacteria (*Alistipes* and *Campylobacter*) in LT ducks at 4 weeks of age, two biomarker bacteria (*Megamonas* and *Butyricicoccus*) in FT ducks at 6 weeks of age, and one biomarker bacterium (*Fusobacteria*) in FT ducks at 4 weeks of age (Figure 7). In addition, we compared species with significant differences between different strains of the same age and different ages of the same strains (Supplementary Figure 1).

### Microbial Functional Characteristics and Prediction Based on Tax4Fun

**FIGURE 8:**
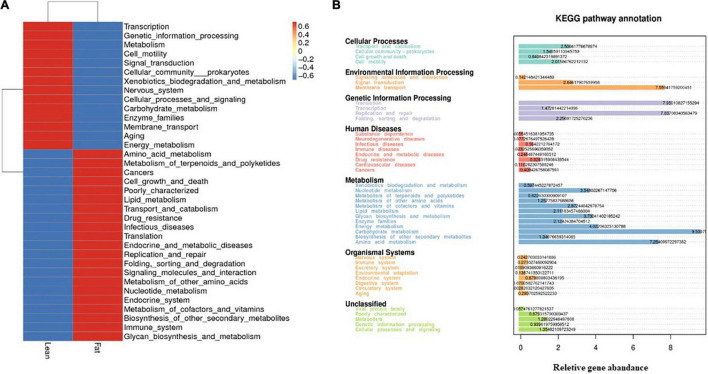
Function Prediction. **(A)** Tax4Fun features annotated clustering heat maps. According to the functional annotations and abundance information of the samples in the database, the top 35 functions and their abundance information in each sample were selected to draw a heat map, and the clustering was conducted from the level of functional differences. **(B)** KEGG pathway annotation. Statistical map of genetic prediction results.

To further understand the metabolic profile of the microbial community, we analyzed the metagenomic functions of bacteria. The top 35 functions based on functional annotations were ranked for each sample and selected for heat map generation. Clustering was conducted based on the level of functional differences (Figure 8A). Metabolic functions in LT cecal microbiota were mainly concentrated in metabolism, xenobiotic biodegradation and metabolism, carbohydrate metabolism, and energy metabolism, whereas FT cecal microbiota were more closely associated with amino acid metabolism, lipid metabolism, transport and catabolism, metabolism of other amino acids, nucleotide metabolism, endocrine system function, metabolism of cofactors and vitamins, biosynthesis of other secondary metabolites, and glycan biosynthesis and metabolism. To further predict how the enriched bacteria promote host phenotypic differences between the two groups, TAX4Fun (Aßhauer et al., 2015) function prediction analysis was performed. KEGG pathway annotation showed that the genes were mainly concentrated in seven categories: cellular processes, environmental information processing, genetic information processing, human diseases, metabolism, organizational systems, and unclassified (Figure 8B). Particularly, carbohydrate metabolism and amino acid metabolism were the most concentrated and closely related to growth and fat generation.

### Correlation Between Performance Indices and Gut Microbiota Diversity

**FIGURE 9:**
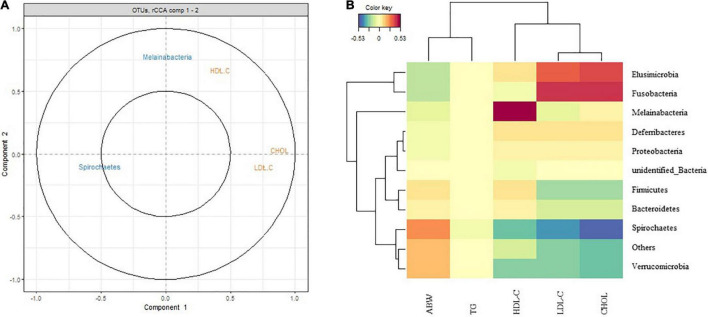
Correlation between performances indices and gut microbiota diversity. **(A)** Correlation Circle plot representing each type of selected features for the Peking duck. The correlation between two variables is positive if the angle is sharp, negative if the angle is obtuse, and null if the vectors are perpendicular. The longer the distance to the origin, the stronger the relationship between the variables. For the sake of interpretability, variables are not represented as vectors but as the end points of the vectors in MixOmics. Two circles are usually represented, of radii 0.5 and 1, to better visualize the “important” variables. **(B)** Clustered Image Map (Euclidean Distance, Complete linkage). Samples are represented in rows, selected features on the first component in columns.

The plot shown in Figure 9 depicts the correlation between performance indices and the gut microbiota. Variable vectors in close proximity to one another are highly correlated. An acute angle between vectors connected by an acute angle (less than 90°) indicates a positive correlation. An obtuse angle (greater than 90°) indicates a negative correlation. A right angle indicates a correlation of zero (Rohart et al., 2017). Thus, the correlation between two features is equal to the cosine of the angle between their vectors (which begin at the origin). Melainabacteria was positively correlated with HDL-C, negatively correlated with LDL-C, and not correlated with CHOL levels. Spirochaetes was negatively correlated with HDL-C, LDL-C, and CHOL levels (Figure 9A). The clustered image map displayed a good correlation between the performance indices and gut microbiota diversity. ABW was positively correlated with Spirochaetes. TG had little correlation with various bacteria. LDL-C was positively correlated with Elusimicrobia and Fusobacteria and negatively correlated with Spirochaetes. CHOL levels were positively correlated with Elusimicrobia and Fusobacteria (Figure 9B).

## Discussion

In recent decades, continuous genetic selection has been used to breed FT and LT Pekin ducks in China, resulting in significant differences in meat production, sebum, and abdominal fat content (Zheng et al., 2014). FT ducks have higher abdominal fat and sebum percentages, and LT ducks have a higher breast muscle percentage. A previous study demonstrated that obese and lean humans have different gut microbiota (DiBaise et al., 2008). Thus, we compared the differences in growth performance, plasma biochemical indices, and intestinal microbial diversity between LT and FT Pekin ducks to investigate the effects of cecal microbiota on fat deposition.

We fed LT and FT ducks with the same commercial feed from 4 to 6 weeks of age. The ABW and average daily feed intake of FT Pekin ducks were significantly higher than those of LT ducks. The high average daily feed intake in FT Pekin ducks may explain the higher sebum and abdominal fat percentages. The results of previous studies differed from this study because of the addition of threonine (Jiang et al., 2020). We also found that the abdominal fat percentage and sebum percentage in FT Pekin ducks were significantly higher than those in LT Pekin ducks. A previous study showed that FT ducks have higher abdominal fat and sebum percentages than LT ducks (Zheng et al., 2014). Furthermore, the plasma concentrations of CHOL, HDL-C, LDL-C, and TG were higher in FT ducks than in LT ducks. A previous study demonstrated that FT ducks have a higher LDL-C content than LT ducks (Jiang et al., 2020). FT chickens were also shown to have higher total CHOL, TG, and lipoprotein concentrations than LT ducks (Musa et al., 2007). LDL-C concentrations are positively correlated with lipid deposition and adiposity in female Pekin ducks (Weyer and Pratley, 1999). In addition, obese rats exhibit elevated serum HDL-C levels compared to their lean counterparts (Jeyakumar et al., 2007). The present study showed that FT ducks have a greater ability to store lipids.

The differences in fat deposition between FT and LT ducks may be attributed to cecal microbial diversity, which potentially regulates fat deposition by affecting gene expression. We showed that the alpha diversity of the microbiota increased with age. The abundance of taxa in the cecal microbiota of different strains of Pekin ducks of the same age was significantly different. Previous studies revealed that varieties and strains affect the abundance of the intestinal microbiota (Bian et al., 2016). Accordingly, the difference in the abundance of cecal microbes between FT and LT ducks may explain the fat deposition in FT ducks.

In the present study, the abundance of *Bacteroides*, *Oscillospira*, and *Parabacteroides* was higher in LT ducks than in FT ducks. Previous studies reported higher abundance of *Oscillospira*, *Rikenellaceae*, *Parabacteroides*, and *Bacteroides fragilis* in patients with non-alcoholic fatty liver disease (Edgar, 2013; Del Chierico et al., 2018; Liu et al., 2018). The average abundance of *Parabacteroides* was negatively correlated with BW but positively correlated with the feed conversion ratio. Fats are easy to store, with a low feed conversion ratio and high BW (Hong et al., 2019; Chen and Yu, 2020). The relative abundance of *Oscillospira* significantly decreased in chickens exposed to fungicides, which altered the structure of the gut microbiota and affected the regulatory mechanism of liver lipid metabolism. The results showed that fungicide exposure increased blood lipid parameters and body fat deposition (Kong et al., 2020). Similarly, the abundance of *Oscillospira* decreased in the intestinal tracts of broilers with a high BW (Lee et al., 2020). *Bacteroides* produce short-chain fatty acids by fermenting dietary indigested polysaccharides and pectin. Short-chain volatile fatty acids (SCFAs), such as acetic acid, propionic acid and butyric acid, are produced by microbial fermentation of the sugars released from non-starch polysaccha-rides and are found in the chicken cecal (Meimandipour et al., 2010). The cecal metagenome encodes several fermentation pathways leading to the production of short-chain fatty acids, including some with novel features. A dozen uptake hydrogenases encoded in the metagenome was found and speculate that these provide major hydrogen sinks within this microbial community and might explain the high abundance of several genera within this microbiome, including Campylobacter, Helicobacter and Megamonas (Sergeant et al., 2014). Gut microbiota contains a rich collection of genes encoding enzymes necessary for decomposition of dietary polysaccharides and oligosaccharides, nitrogen metabolism, fatty acid and lipid metabolism, and pathways involved in a hydrogen sink. Poultry, like most animals, lake the genes for glycoside hydrolase, and carbohydrate esterse enzymes that are necessary to facilitate the degradation of non-starch polysaccharides. During the decomposition of dietary polysaccharides, bacteria produce short-chain (volatile) fatty acids (SCFAs), such as acetic, propionic, and butyric acid. These SCFASs are absorbed transepithelial and serve as a source of energy for the host. the accumulation of molecular hydrogen released during fermentation leads to fermentation slowdown or to the production of less energy-efficient substance, such as ethanol. butyrate and propionate. The presence of bacteria that act as a hydrogen sink result in a switch to the more productive fermentation into acetate and increased production of SCFAs. Such activity could lead to a significant improvement in poultry production and the associated economics (Binek et al., 2017).

Short-chain fatty acids can regulate host body energy homeostasis, protect the host from inflammation, and inhibit fat mass development (He et al., 2016). When swine diets were supplemented with Glu + Arg, the colonic butyrate and propionate concentrations and Actinobacteria abundance increased, and body fat weight in finishing swine decreased. Actinobacteria, which are negatively associated with both average and waist backfat, produce many bioactive metabolites, including antibacterial and antiviral agents for growth-promoting substances in plants and animals. Therefore, we predicted that the higher abundance of *Oscillospira*, *Parabacteroides*, and *Bacteroides* would inhibit fat deposition in LT ducks.

Increasing evidence shows that the gut microbiota affects fat metabolism and helps to harvest energy and increase host fat storage (Samuel et al., 2008; Goldsammler et al., 2018). Firmicutes and Bacteroidetes are dominant among the intestinal microorganisms of various animals, such as humans, horses, rabbits, and beef cattle (Larsen et al., 2010; Shanks et al., 2011; Shepherd et al., 2012; Zou et al., 2016). An increased ratio of gram-positive Firmicutes and gram-negative Bacteroidetes can lead to obesity, whereas their decreased ratio can lead to weight loss (Bell, 2015; Mathur and Barlow, 2015; Riva et al., 2017). High levels of Firmicutes and low levels of Bacteroidetes in the gut may increase fat synthesis and storage (Ding et al., 2016). Alterations affecting the dominant phyla, Firmicutes and Bacteroidetes, were first described in obese animals and subjects with increased abundance of Firmicutes at the expense of Bacteroidetes (Ley et al., 2007). When these subjects were fed a calorie-restricted diet for 1 year, the abundance of Bacteroidetes increased and the Firmicutes/Bacteroidetes ratio normalized in parallel with weight loss. These studies were supported by other studies in animals fed high-fat or high-fiber diets showing higher Firmicutes and Bacteroidetes abundance, respectively (de Wit et al., 2012). In this study, these two bacterial phyla were dominant. From 4 to 6 weeks of age, the Firmicutes/Bacteroidetes ratio in FT Pekin ducks showed an increasing trend. However, this ratio showed little change with age in LT Pekin ducks. Therefore, an increase in the ratio of these two bacteria may increase fat deposition in FT Pekin ducks compared to that in LT Pekin ducks. These results indicate that the intestinal microbiome greatly helps the host to obtain energy from food and promotes fat deposition. Large amounts of TGs are transported to the abdomen *via* the blood, leading to the deposition of abdominal fat in Pekin ducks.

Verrucomicrobia, Bacteroidetes, Proteobacteria, and Elusimicrobia were less abundant in patients with both obesity and type 2 diabetes mellitus (Hildebrandt et al., 2009; Ahmad et al., 2019). In the present study, HDL-C was positively correlated with Melainabacteria and negatively correlated with Spirochaetes. LDL-C was positively correlated with Elusimicrobia and Fusobacteria and negatively correlated with Spirochaetes and Melainabacteria. CHOL levels were positively correlated with Elusimicrobia and Fusobacteria and negatively correlated with Spirochaetes. ABW was positively correlated with Spirochaetes. The correlation between the above-mentioned bacteria and fat deposition indicates the regulatory role of these bacteria in fat deposition, which should be confirmed in further studies.

## Conclusion

In conclusion, our results showed that FT ducks had higher percentages of sebum and abdominal fat than LT ducks. These positive effects may be associated with the altered gut bacterial microbiota, particularly an increased Firmicutes/Bacteroidetes ratio and reduced cecal abundance of Oscillospira, Parabacteroides, and Bacteroides in FT ducks, which are known to affect lipid deposition.

## Data Availability Statement

The original contributions presented in the study are publicly available. This data can be found here: https://www.ncbi.nlm.nih.gov/sra/, SRP347918.

## Ethics Statement

The animal study was reviewed and approved by the Animal Management Committee (in charge of animal welfare issues) of the Institute of Animal Science, Chinese Academy of Agricultural Sciences (IAS-CAAS, Beijing, China).

## Author Contributions

TY, SH, and GChe contributed to the conception and design of the work, drafted the work, and substantively revised it. YJ revised the manuscript substantively. TY, GCha, and WZ performed the experiments. JT analyzed the data. All authors have read and approved the final manuscript.

## Conflict of Interest

The authors declare that the research was conducted in the absence of any commercial or financial relationships that could be construed as a potential conflict of interest.

## Publisher’s Note

All claims expressed in this article are solely those of the authors and do not necessarily represent those of their affiliated organizations, or those of the publisher, the editors and the reviewers. Any product that may be evaluated in this article, or claim that may be made by its manufacturer, is not guaranteed or endorsed by the publisher.

## Supplementary Material

Supplementary Figure 1 Beta Diversity LEfSe. Pairwise comparison of different strains of the same strain at different age and different strains of the same age. Histogram of the LDA scores computed for spieces differentially abundant between different strains and different ages groups. The LDA score (>2 considered) indicates the effect size and ranking of each differentially abundant taxon. L.WK4, Four-week-old lean-type Pekin duck; F.WK4, Four-week-old fatty-type Pekin duck; L.WK6, Six-week-old lean-type Pekin duck; F.WK6, Six-week-old fatty-type Pekin duck.
