# Supplementary material for: Comparison of Cecal Microbiota and Performance Indices Between Lean-Type and Fatty-Type Pekin Ducks
Source: Front Microbiol. 2022 Mar 8;13:820569. doi: 10.3389/fmicb.2022.820569 (PMC8957900; doi:10.3389/fmicb.2022.820569)
Supplement: Supplementary file 1 [file Data_Sheet_1.docx]

**Supplement Table 1.** The top 10 bacteria at phylum level of the relative abundance values and P-values

| Bacteria | Group | Average | SD | P-value | |
| --- | --- | --- | --- | --- | --- |
| Firmicutes | L.WK4 | 0.31119 | 0.04853 | L.WK6 | 0.814 |
|  |  |  |  | F.WK4 | 0.114 |
|  | F.WK4 | 0.25133 | 0.07434 | L.WK4 | 0.114 |
|  |  |  |  | F.WK6 | 0.002 |
|  | L.WK6 | 0.30269 | 0.06376 | L.WK4 | 0.814 |
|  |  |  |  | F.WK6 | 0.055 |
|  | F.WK6 | 0.37402 | 0.11731 | L.WK6 | 0.055 |
|  |  |  |  | F.WK4 | 0.002 |
| Bacteroidetes | L.WK4 | 0.45717 | 0.07753 | L.WK6 | 0.766 |
|  |  |  |  | F.WK4 | 0.730 |
|  | F.WK4 | 0.44135 | 0.07313 | L.WK4 | 0.730 |
|  |  |  |  | F.WK6 | 0.466 |
|  | L.WK6 | 0.47041 | 0.09913 | L.WK4 | 0.766 |
|  |  |  |  | F.WK6 | 0.166 |
|  | F.WK6 | 0.40793 | 0.13177 | L.WK6 | 0.166 |
|  |  |  |  | F.WK4 | 0.466 |
| Fusobacteria | L.WK4 | 0.03570 | 0.03563 | L.WK6 | 0.754 |
|  |  |  |  | F.WK4 | 0.003 |
|  | F.WK4 | 0.15881 | 0.16243 | L.WK4 | 0.003 |
|  |  |  |  | F.WK6 | 0.004 |
|  | L.WK6 | 0.04769 | 0.04195 | L.WK4 | 0.754 |
|  |  |  |  | F.WK6 | 0.787 |
|  | F.WK6 | 0.03734 | 0.04001 | L.WK6 | 0.787 |
|  |  |  |  | F.WK4 | 0.004 |
| Proteobacteria | L.WK4 | 0.13171 | 0.06292 | L.WK6 | 0.411 |
|  |  |  |  | F.WK4 | 0.514 |
|  | F.WK4 | 0.11355 | 0.05279 | L.WK4 | 0.774 |
|  |  |  |  | F.WK6 | 0.514 |
|  | L.WK6 | 0.10942 | 0.06810 | L.WK4 | 0.887 |
|  |  |  |  | F.WK6 | 0.411 |
|  | F.WK6 | 0.10559 | 0.05390 | L.WK6 | 0.774 |
|  |  |  |  | F.WK4 | 0.877 |
| unidentified Bacteria | L.WK4 | 0.02498 | 0.07368 | L.WK6 | 0.236 |
|  |  |  |  | F.WK4 | 0.449 |
|  | F.WK4 | 0.01091 | 0.01373 | L.WK4 | 0.449 |
|  |  |  |  | F.WK6 | 0.924 |
|  | L.WK6 | 0.00339 | 0.00512 | L.WK4 | 0.236 |
|  |  |  |  | F.WK6 | 0.750 |
|  | F.WK6 | 0.00914 | 0.02462 | L.WK6 | 0.750 |
|  |  |  |  | F.WK4 | 0.924 |
| Spirochaetes | L.WK4 | 0.00093 | 0.00226 | L.WK6 | 0.005 |
|  |  |  |  | F.WK4 | 0.963 |
|  | F.WK4 | 0.00017 | 0.00016 | L.WK4 | 0.963 |
|  |  |  |  | F.WK6 | 0.350 |
|  | L.WK6 | 0.04754 | 0.06569 | L.WK4 | 0.005 |
|  |  |  |  | F.WK6 | 0.048 |
|  | F.WK6 | 0.01544 | 0.02167 | L.WK6 | 0.048 |
|  |  |  |  | F.WK4 | 0.350 |
| Deferribacteres | L.WK4 | 0.03213 | 0.02806 | L.WK6 | 0.164 |
|  |  |  |  | F.WK4 | 0.238 |
|  | F.WK4 | 0.01400 | 0.01038 | L.WK4 | 0.238 |
|  |  |  |  | F.WK6 | 0.050 |
|  | L.WK6 | 0.01123 | 0.01063 | L.WK4 | 0.164 |
|  |  |  |  | F.WK6 | 0.029 |
|  | F.WK6 | 0.04466 | 0.05663 | L.WK6 | 0.029 |
|  |  |  |  | F.WK4 | 0.050 |
| Elusimicrobia | L.WK4 | 0.00158 | 0.00125 | L.WK6 | 0.638 |
|  |  |  |  | F.WK4 | 0.023 |
|  | F.WK4 | 0.00532 | 0.00530 | L.WK4 | 0.023 |
|  |  |  |  | F.WK6 | 0.051 |
|  | L.WK6 | 0.00085 | 0.00072 | L.WK4 | 0.638 |
|  |  |  |  | F.WK6 | 0.408 |
|  | F.WK6 | 0.00214 | 0.00434 | L.WK6 | 0.408 |
|  |  |  |  | F.WK4 | 0.051 |
| Verrucomicrobia | L.WK4 | 0.00001 | 0.00003 | L.WK6 | 0.107 |
|  |  |  |  | F.WK4 | 0.999 |
|  | F.WK4 | 0.00001 | 0.00003 | L.WK4 | 0.999 |
|  |  |  |  | F.WK6 | 0.998 |
|  | L.WK6 | 0.00195 | 0.00517 | L.WK4 | 0.107 |
|  |  |  |  | F.WK6 | 0.107 |
|  | F.WK6 | 0.00001 | 0.00002 | L.WK6 | 0.107 |
|  |  |  |  | F.WK4 | 0.998 |
| Melainabacteria | L.WK4 | 0.00214 | 0.00192 | L.WK6 | 0.831 |
|  |  |  |  | F.WK4 | 0.547 |
|  | F.WK4 | 0.00274 | 0.00275 | L.WK4 | 0.547 |
|  |  |  |  | F.WK6 | 0.075 |
|  | L.WK6 | 0.00193 | 0.00206 | L.WK4 | 0.831 |
|  |  |  |  | F.WK6 | 0.304 |
|  | F.WK6 | 0.00092 | 0.00187 | L.WK6 | 0.304 |
|  |  |  |  | F.WK4 | 0.075 |

L.WK4, Four-week-old lean-type Pekin duck; F.WK4, Four-week-old fatty-type Pekin duck; L.WK6, Six-week-old lean-type Pekin duck; F.WK6, Six-week-old fatty-type Pekin duck.
