# Supplementary material for: Comparison of Cecal Microbiota and Performance Indices Between Lean-Type and Fatty-Type Pekin Ducks
Source: Front Microbiol. 2022 Mar 8;13:820569. doi: 10.3389/fmicb.2022.820569 (PMC8957900; doi:10.3389/fmicb.2022.820569)
Supplement: Supplementary file 2 [file Data_Sheet_2.docx]

**Supplement Table 2.** The top 10 bacteria at genus level of the relative abundance values and P-values

| genus horizontal relative abundance | Group | Average | SD | P-value | |
| --- | --- | --- | --- | --- | --- |
| Bacteroides | L.WK4 | 0.2873 | 0.0680 | L.WK6 | 0.331 |
|  |  |  |  | F.WK4 | 0.320 |
|  | F.WK4 | 0.3219 | 0.0528 | L.WK4 | 0.320 |
|  |  |  |  | F.WK6 | 0.381 |
|  | L.WK6 | 0.2544 | 0.0817 | L.WK4 | 0.331 |
|  |  |  |  | F.WK6 | 0.534 |
|  | F.WK6 | 0.2820 | 0.1102 | L.WK6 | 0.534 |
|  |  |  |  | F.WK4 | 0.381 |
| Fusobacterium | L.WK4 | 0.0357 | 0.0356 | L.WK6 | 0.770 |
|  |  |  |  | F.WK4 | 0.006 |
|  | F.WK4 | 0.1587 | 0.1625 | L.WK4 | 0.006 |
|  |  |  |  | F.WK6 | 0.021 |
|  | L.WK6 | 0.0477 | 0.0419 | L.WK4 | 0.770 |
|  |  |  |  | F.WK6 | 0.681 |
|  | F.WK6 | 0.0254 | 0.0171 | L.WK6 | 0.681 |
|  |  |  |  | F.WK4 | 0.021 |
| Desulfovibrio | L.WK4 | 0.0912 | 0.0583 | L.WK6 | 0.428 |
|  |  |  |  | F.WK4 | 0.348 |
|  | F.WK4 | 0.0657 | 0.0362 | L.WK4 | 0.348 |
|  |  |  |  | F.WK6 | 0.776 |
|  | L.WK6 | 0.0703 | 0.0771 | L.WK4 | 0.428 |
|  |  |  |  | F.WK6 | 0.674 |
|  | F.WK6 | 0.0557 | 0.0327 | L.WK6 | 0.674 |
|  |  |  |  | F.WK4 | 0.776 |
| Campylobacter | L.WK4 | 0.0247 | 0.0732 | L.WK6 | 0.200 |
|  |  |  |  | F.WK4 | 0.465 |
|  | F.WK4 | 0.0106 | 0.0137 | L.WK4 | 0.465 |
|  |  |  |  | F.WK6 | 0.698 |
|  | L.WK6 | 0.0004 | 0.0004 | L.WK4 | 0.200 |
|  |  |  |  | F.WK6 | 0.987 |
|  | F.WK6 | 0.0008 | 0.0004 | L.WK6 | 0.987 |
|  |  |  |  | F.WK4 | 0.698 |
| Brachyspira | L.WK4 | 0.0009 | 0.0023 | L.WK6 | 0.008 |
|  |  |  |  | F.WK4 | 0.965 |
|  | F.WK4 | 0.0002 | 0.0002 | L.WK4 | 0.965 |
|  |  |  |  | F.WK6 | 0.590 |
|  | L.WK6 | 0.0475 | 0.0657 | L.WK4 | 0.008 |
|  |  |  |  | F.WK6 | 0.117 |
|  | F.WK6 | 0.0122 | 0.0145 | L.WK6 | 0.117 |
|  |  |  |  | F.WK4 | 0.590 |
| Mucispirillum | L.WK4 | 0.0321 | 0.0281 | L.WK6 | 0.147 |
|  |  |  |  | F.WK4 | 0.219 |
|  | F.WK4 | 0.0140 | 0.0104 | L.WK4 | 0.219 |
|  |  |  |  | F.WK6 | 0.020 |
|  | L.WK6 | 0.0112 | 0.0106 | L.WK4 | 0.147 |
|  |  |  |  | F.WK6 | 0.013 |
|  | F.WK6 | 0.0603 | 0.0809 | L.WK6 | 0.013 |
|  |  |  |  | F.WK4 | 0.020 |
| Butyricicoccus | L.WK4 | 0.0230 | 0.0109 | L.WK6 | 0.751 |
|  |  |  |  | F.WK4 | 0.224 |
|  | F.WK4 | 0.0108 | 0.0033 | L.WK4 | 0.224 |
|  |  |  |  | F.WK6 | <0.001 |
|  | L.WK6 | 0.0261 | 0.0091 | L.WK4 | 0.751 |
|  |  |  |  | F.WK6 | <0.001 |
|  | F.WK6 | 0.0811 | 0.0617 | L.WK6 | <0.001 |
|  |  |  |  | F.WK4 | <0.001 |
| Erysipelatoclostridium | L.WK4 | 0.0039 | 0.0023 | L.WK6 | 0.233 |
|  |  |  |  | F.WK4 | 0.932 |
|  | F.WK4 | 0.0029 | 0.0020 | L.WK4 | 0.932 |
|  |  |  |  | F.WK6 | 0.782 |
|  | L.WK6 | 0.0173 | 0.0440 | L.WK4 | 0.233 |
|  |  |  |  | F.WK6 | 0.487 |
|  | F.WK6 | 0.0070 | 0.0053 | L.WK6 | 0.487 |
|  |  |  |  | F.WK4 | 0.782 |
| Megamonas | L.WK4 | 0.0341 | 0.0306 | L.WK6 | 0.008 |
|  |  |  |  | F.WK4 | 0.873 |
|  | F.WK4 | 0.0357 | 0.0247 | L.WK4 | 0.873 |
|  |  |  |  | F.WK6 | 0.744 |
|  | L.WK6 | 0.0054 | 0.0023 | L.WK4 | 0.008 |
|  |  |  |  | F.WK6 | 0.060 |
|  | F.WK6 | 0.0313 | 0.0201 | L.WK6 | 0.060 |
|  |  |  |  | F.WK4 | 0.744 |
| Alistipes | L.WK4 | 0.0381 | 0.0309 | L.WK6 | 0.004 |
|  |  |  |  | F.WK4 | 0.154 |
|  | F.WK4 | 0.0231 | 0.0265 | L.WK4 | 0.154 |
|  |  |  |  | F.WK6 | 0.264 |
|  | L.WK6 | 0.0066 | 0.0030 | L.WK4 | 0.004 |
|  |  |  |  | F.WK6 | 0.917 |
|  | F.WK6 | 0.0079 | 0.0035 | L.WK6 | 0.917 |
|  |  |  |  | F.WK4 | 0.264 |

L.WK4, Four-week-old lean-type Pekin duck; F.WK4, Four-week-old fatty-type Pekin duck; L.WK6, Six-week-old lean-type Pekin duck; F.WK6, Six-week-old fatty-type Pekin duck.
