# Supplementary figures and images for: Comparison of Cecal Microbiota and Performance Indices Between Lean-Type and Fatty-Type Pekin Ducks
Source: Front Microbiol. 2022 Mar 8;13:820569. doi: 10.3389/fmicb.2022.820569 (PMC8957900; doi:10.3389/fmicb.2022.820569)

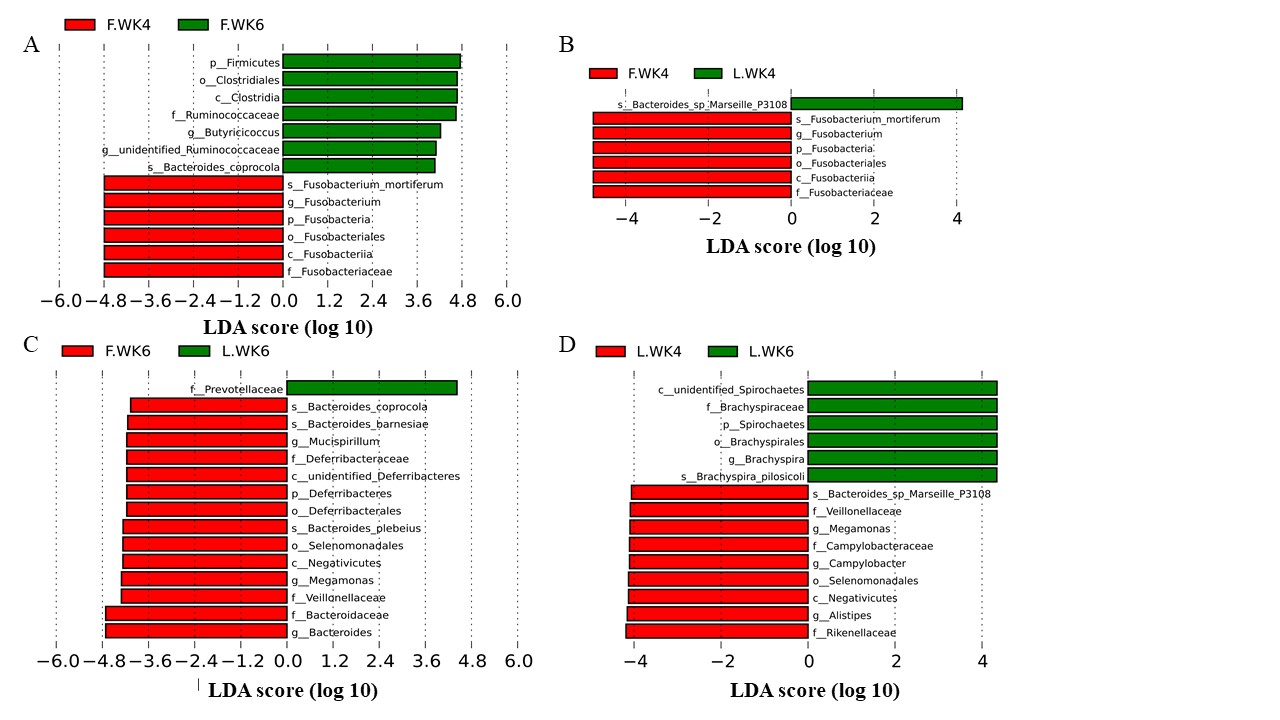

Supplement: Supplementary Figure 1 — Beta Diversity LEfSe. Pairwise comparison of different strains of the same strain at different age and different strains of the same age. Histogram of the LDA scores computed for spieces differentially abundant between different strains and different ages groups. The LDA score (>2 considered) indicates the effect size and ranking of each differentially abundant taxon. L.WK4, Four-week-old lean-type Pekin duck; F.WK4, Four-week-old fatty-type Pekin duck; L.WK6, Six-week-old lean-type Pekin duck; F.WK6, Six-week-old fatty-type Pekin duck. [file Image_1.JPEG]
